# Heterologous Expression of Mannanase and Developing a New Reporter Gene System in *Lactobacillus casei* and *Escherichia coli*

**DOI:** 10.1371/journal.pone.0142886

**Published:** 2015-11-12

**Authors:** Jinzhong Lin, Yexia Zou, Chengjie Ma, Qunxin She, Yunxiang Liang, Zhengjun Chen, Xiangyang Ge

**Affiliations:** 1 State Key Laboratory of Agricultural Microbiology, Huazhong Agricultural University, Wuhan, China; 2 State Key Laboratory of Dairy Biotechnology, Technology Center of Bright Dairy & Food Co., Ltd., 1518 Jiangchang Road (W), Shanghai, 200436, China; 3 Department of Biology, University of Copenhagen, Biocenter, Ole Maaloes Vej 5, DK-2200, Copenhagen N, Denmark; University of Houston, UNITED STATES

## Abstract

Reporter gene systems are useful for studying bacterial molecular biology, including the regulation of gene expression and the histochemical analysis of protein products. Here, two genes, β-1,4-mannanase (*manB*) from *Bacillus pumilus* and β-glucuronidase (*gusA*) from *Escherichia coli* K12, were cloned into the expression vector pELX1. The expression patterns of these reporter genes in *Lactobacillus casei* were investigated by measuring their enzymatic activities and estimating their recombinant protein yields using western blot analysis. Whereas mannanase activity was positively correlated with the accumulation of ManB during growth, GusA activity was not; western blot analysis indicated that while the amount of GusA protein increased during later growth stages, GusA activity gradually decreased, indicating that the enzyme was inactive during cell growth. A similar trend was observed in *E*. *coli* JM109. We chose to use the more stable mannanase gene as the reporter to test secretion expression in *L*. *casei*. Two pELX1-based secretion vectors were constructed: one carried the signal peptide of the unknown secretion protein Usp45 from *Lactococcus lactis* (pELSH), and the other contained the full-length SlpA protein from the S-layer of *L*. *acidophilus* (pELWH). The secretion of ManB was detected in the supernatant of the pELSH-ManB transformants and in the S-layer of the cell surface of the pELWH-ManB transformants. This is the first report demonstrating that the *B*. *pumilus manB* gene is a useful reporter gene in *L*. *casei* and *E*.*coli*.

## Introduction

Lactic acid bacteria (LAB) utilize a mixed variety of sugars to produce lactic acid by facultative anaerobic fermentation. These bacteria are widespread in various natural environments including foods, fermentation products, human and animal gastrointestinal tracts and plant surfaces. LAB have served as traditional industrial bacteria and are widely used in the processing of various foods, such as dairy, meat and pickles [1]. Furthermore, LAB represent one of the dominant groups of microorganisms in the human gastrointestinal tract and play important roles in this ecosystem, including improving lactose digestion, reducing gastrointestinal disorders, and enhancing cellular immunity, thereby facilitating the cure of some human diseases [2,3]. For these reasons, some LAB strains have been used as probiotic bacteria [4]. LAB are organisms generally regarded as safe and have been used widespread in food and feedstock industries, and LAB have the potential prospect to be live vehicles for mucosal delivery of health promoting molecules [5–7].

Since the 1990s, much research has been devoted to developing gene expression systems for LAB. *Lactococcus* (especially *Lc*. *lactis*), *Lactobacillus* and *Streptococcus* have all been developed into useful cell factories for producing recombinant proteins [8]. However, the known LAB protein expression systems usually confer only a low level of recombinant protein expression. In order to test the efficiency of different LAB expression systems, a suitable reporter gene system is required.

Several genes are used as reporter genes in LAB bacterial genetics, such as those encoding chloramphenicol acetyltransferase (*cat-194* from *Staphylococcus aureus* and *cat-86* from *B*. *pumilus*) [9,10], the *B*. *licheniformis* α-amylase [11], the *Vibrio fischeri* luciferase [12], the *E*. *coli* β-glucuronidase [13], the *Leuconostoc mesenteroides* β-galactosidase [14], and the *S*. *aureus* nuclease [15]. Moreover, green fluorescent protein (*GFP*) from the jellyfish *Aequorea victoria* [16] has been exploited as a reporter and used successfully in a variety of bacteria including different LAB organisms [17–19].

Here, we investigated the usefulness of a known reporter gene, the *E*. *coli* β-glucuronidase gene, and another heterologous gene, the *B*. *pumilus* β-1,4-mannanase gene, in *Lactobacillus casei* using the *L*. *casei* expression vector pELX1 derived from the naturally occurring pMC11 plasmid [20]. Furthermore, the signal peptide for the secreted protein Usp45 from *Lc*. *lactis* [21] and the full-length SlpA protein from the S-layer of *L*. *acidophilus* NCFM [22] were tested for their capacity to drive the secretion of heterologous proteins in *L*. *casei*. Our results indicate that whereas the β-1,4-mannanase is a highly stable enzyme, the β-glucuronidase protein was gradually inactivated during bacterial growth.

## Materials and Methods

### Bacterial strains and growth conditions

The bacterial strains and plasmids used in this study are listed in Table 1. *L*. *casei* strains were grown in MRS broth (OXOID, Hampshire, England) at 30°C without shaking. *E*. *coli* JM109 was propagated in Luria-Bertani medium (Thermo Scientific Molecular Biology, Waltham, MA, USA) at 37°C with moderate shaking. Antibiotics were used as follows: ampicillin (100 μg·ml^-1^, Sigma, St. Louis, MO, USA) for *E*. *coli* strains and erythromycin (10 μg·ml^-1^, Sigma) for *L*. *casei* strains.

**Table 1:** Bacterial strains and plasmids utilized in this study.

Strain or plasmid	Description	Reference
**Strains**		
*L*. *casei* MCJΔ1	Transformation/expression host, a pMC11 plasmid-free derivative of *L*. *casei* MCJ (CCTCC AB2013356)	(20)
*B*. *pumilus*	β-1,4-mannanase gene (*manB*, GenBank No. AEO79931.1), 1104 bp	CCTCC AB94044
*L*. *acidophilus* NCFM	*slpA* gene (GenBank No. AAV42070.1), 1335 bp	ATCC SD5221
*Lc*. *lactis* subsp. *lactis* MG1363	Secreted protein Usp45 signal peptide (SP_Usp45_) (GenBank No. M60178.1)	(22)
*E*. *coli* K12	β-glucuronidase gene (*gusA*, GenBank No. AAA68923.1), 1809 bp	NEB (USA)
*E*. *coli* JM109	Transformation/expression host	Takara (Japan)
**Plasmid**		
pELX1	Cloning/expression vector	(20)
pELSH	SP_Usp45_ was cloned into pELX1	This work
pELWH	*slpA* was cloned into pELX1	This work
pELX1-ManB	*manB* as a reporter gene cloned into pLEX1	This work
pELSH-ManB	*manB* as a reporter gene cloned into pELSH	This work
pELWH-ManB	*manB* as a reporter gene cloned into pELWH	This work
pELX1-GusA	*gusA* as a reporter gene cloned into pLEX1	This work

### DNA Manipulations

Standard molecular cloning techniques were performed [23]. Total DNA was extracted from *Lactobacillus* cells according to a previously described method [20]. Plasmid DNA was isolated from *E*. *coli* using an Omega Plasmid Mini-Preparation Kit (OMEGA bio-tek, Norcross, GA, USA), and PCR products were purified with an Omega Cycle-Pure Kit (OMEGA bio-tek, Norcross, GA, USA). Pfu and Taq polymerases were purchased from Transgen (Beijing Transgen Co. Ltd., Beijing, China), and DNA restriction enzymes and T4 DNA ligase were purchased from Fermentas (Thermo Scientific Molecular Biology). All enzymes were used according to the manufacturer’s instructions.

### Construction of plasmids for recombinant protein expression

DNA fragment encoding the mature peptide of *B*. *pumilus manB* was PCR amplified with the primers *Nco*I-*manB*-F and *Kpn*I-*manB*-R (Table 2) using the total DNA extracted from *B*. *pumilus* as template. The PCR product was digested with the restriction enzymes (REs) *Nco*I and *Kpn*I and ligated to plasmid vector pELX1 pre-linearized with the same REs, yielding pELX1-ManB. Using the same strategy, the *E*. *coli gusA* was obtained by PCR, and the resulting PCR product was cloned into pELX1 to generate the other expression plasmid, pELX1-GusA.

**Table 2:** Primers utilized in this study.

Primer name	Sequence (5’ →3’)
*Nco*I-*manB*-F	CATGCCATGGCATACTGTGTCGCCTGTGAATC
*Kpn*I-*manB*-R	CCGGGGTACCTCATTCAACGATTGGCGTTAAAG
*Nco*I-*gusA*-F	CATGCCATGGATGTTACGTCCTGTAGAAACCC
*Kpn*I-*gusA*-R	CCGGGGTACCTCATTGTTTGCCTCCCTGCTGC
*Eco*RI*-*P_*SlpA*_-F	CCGGGAATTCAAGCGGTAGGTGAAATATTAC
P_*SlpA*_-R	CATGTGGTCTTTTCCTCCTTG
*Xho*I-MCS-F	GGCCCTCGAGCACCATCATCATCATCATGAC
*Bam*HI*-*T*-*R	GGCCGGATCCAGCTTGCGTTTGATTTTC
SOE- SP_Usp45_-F	CAAGGAGGAAAAGACCACATGAAAAAAAAGATTATCTCAGCTA
*Xho*I-SP_Usp45_-R	GGCCCTCGAGGTTTGTGTCAGCGTAAACACC
*Xho*I-*SlpA*pro-R	GGCCCTCGAGTCTAAAGTTTGCAACCTTAACG

For protein secretion, the DNA fragment encoding the signal peptide of secreted protein Usp45 in *Lc*. *lactis* was amplified using the primers SOE-SP_Usp45_-F and XhoI-SP_Usp45_-R. The promoter fragment of the *L*. *acidophilus* S-layer protein precursor-encoding gene (P_*SlpA*_) was amplified with the primers *Eco*RI*-*P_*SlpA*_-F and P_*SlpA*_-R. Then, the two PCR products were fused together by splicing by overlap extension PCR [24]. The resulting fragment was then digested with *Eco*RI and *Xho*I. The DNA fragment including a sequence coding for a 6×His-tag, a multiple cloning site and a transcriptional terminator was amplified from plasmid pELX1 using the primers *Xho*I-MCS-F and *Bam*HI*-*T-R and subsequently digested with *Bam*HI and *Xho*I. After the digestion of pEL5.6 (Table 1) with *Eco*RI and *Bam*HI, the three DNA fragments were ligated together in a triple ligation, yielding the secreted expression vector pELSH. To construct the secretion vector, the full-length *L*. *acidophilus slpA* gene with native promoter, was amplified from *L*. *acidophilus* NCFM genomic DNA using the primers *Eco*RI*-*P_*SlpA*_-F and *Xho*I-*SlpA*pro-R. The resulting DNA fragment was digested with *Eco*RI and *Xho*I and inserted into pELSH at the *Eco*RI and *Xho*I sites, generating the secretion expression vector pELWH. Finally, the *manB* gene was cloned into the vectors pELSH and pELWH using the same strategy as for pELX1-ManB, yielding pELSH-ManB and pELWH-ManB, respectively. All constructs were verified by restriction analysis and sequencing.

### Electroporation transformation

Electroporation transformation of *L*. *casei* was performed according to previously reported [20] using a Multiporator (Eppendorf, Hamburg, Germany) and the transformants were screened on MRS agar plates containing erythromycin.

### Detection of target proteins in lactobacilli cells

For intracellular expression, 500 ml MRS broth was inoculated with a *L*. *casei* transformant culture grown overnight (1%, v/v), 20 ml fractions of the culture were taken every 1 hours to measure the OD_600_ (optical density at 600 nm), and cells were then collected by centrifugation at 12000 rpm for 5 min. Whole cell proteins (WCP) were prepared from cells of *L*. *casei* transformants using a high-pressure homogenizer (Siemens, Munich, Germany) and then the WCP was determined for ManB or GusA activity and analyzed by sodium dodecyl sulfate-polyacrylamide gel electrophoresis (SDS-PAGE) by an electrophoresis system (Bio-Rad, Hercules, CA, USA), which was performed on a 10% (w/v) running gel. For secretory expression, cells were collected by centrifugation at 12000 rpm for 5 min, and the supernatant was filtered by 0.22 μm filter membrane, then the effluent was determined for ManB activity and western blot analysis.

For western blot analysis, the procedure was performed according to previously reported [20], the primary antibody was mouse antibody against His-tag (Beyotime, Shanghai, China) and the second antibody was HRP-labeled goat antibody IgG against mouse IgG (Goat Anti-Mouse IgG/HRP, Beijing Biosynthesis Biotechnology Co. Ltd., Beijing, China), the membrane was stained with the HRP Substrate (Western ECL Substrate, Bio-Rad) to reveal the presence of the target proteins.

### Enzyme assay

β-1,4-mannanase activity was determined using the substrate glucomannan, which was purified from tubers of the traditional food plant *Amorphophallus konjac* (Kevin Food Co. Ltd., Guangzhou, China). The reaction mixture contained 2 ml of a diluted enzyme sample and 2 ml of a 0.5% (w/v) solution of the substrate in 0.1 M sodium acetate buffer (pH 5.5), both of which were pre-warmed to 50°C. The mixture was then incubated for 30 min, and the reaction was stopped by adding 5 ml of 3, 5-dinitrosalicylic acid (DNS) [25]. After boiled for 10 min, the final volume was adjusted to 25 ml, and the concentration of the mannose-oligosaccharides reducing ends was estimated by measuring the absorbance at 540 nm using a spectrophotometer DU730 (Beckman, Brea, CA, USA). Serving as a negative control, another sample was prepared identically except the enzyme was added to the reaction after the DNS reagent. For S-layer-bound mannanase activity, a culture aliquot containing 1×10^10^ cfu was applied as crude extract in each reaction tube. One unit of enzyme activity is defined as the amount of enzyme needed to liberate reducing sugars equivalent to 1 μmol of D-mannose in 1 min at 50°C at pH 5.5.

β-glucuronidase activity was determined as previously described [13] using 4-nitrophenyl-β-D-glucuronide (Sigma) as the substrate. The reaction mixture containing 0.5 ml of the enzyme sample and 0.5 ml of a 1 mM 4-nitrophenyl-β-D-glucuronide solution in GUS buffer (0.05 M sodium phosphate [pH 7.0], 10 mM β-mercaptoethanol, 1 mM EDTA [ethylene diamine tetra-acetic acid], 0.1% (v/v) Triton X-100) was prepared and incubated at 37°C for 10 min. After incubation, the reaction was stopped by adding 0.1 ml of 0.4 M sodium hydroxide. The enzyme activity was estimated by measuring the absorbance of the sample at 420 nm using a microplate reader (Bio-Tek, Winooski, VT, USA). Reaction rates were quantified using 4-nitrophenol release (with a molar extinction coefficient of 18,400 L·mol^-1^·cm^-1^ at OD_401_). One unit of enzyme activity is defined as the amount of enzyme necessary to liberate 1 nmol of 4-nitrophenol in 1 min at 37°C at pH 7.0.

The concentration of WCP was measured according to the Bradford method [26] using the Bio-Rad protein assay with bovine serum albumin as the standard.

### S-layer protein electrophoresis

Extracellular proteins were extracted from *Lactobacillus* cells on the method as described by Hagen et al. [27]. Samples of the S-layer proteins were prepared as follows: a total of 2 ml of *L*. *casei* transformants were centrifuged at 12,000 rpm for 2 min, washed twice with 2 ml PBS buffer, resuspended in 80 μl double-distilled water, and then mixed with 20 μl 5× Laemmli sample buffer (300 mM Tris-HCl [pH 6.8], 10% (w/v) sodium dodecyl sulfate, 50% (v/v) glycerol, 25% (v/v) β-mercaptoethanol, 0.05% (w/v) bromophenol blue). After boiling for 10 min, the mixture was centrifuged at 5,000 rpm for 5 min, and 20 μl sample of the supernatant (the cell surface-associated proteins) was analyzed by SDS-PAGE and western blot.

### Statistical analysis

All physicochemical experiments were carried out in triplicate and each value was calculated with the mean and standard deviation. Statistical analysis was performed using Microsoft Excel 2010 (Redmond, WA, USA).

## Results

### *L*. *acidophilus* P_*SlpA*_ mediates protein expression in both *E*. *coli* and *L*. *casei*

Previously, we showed that the *slpA* promoter of *L*. *acidophilus* (P_*SlpA*_) allowed expression of the enhanced green fluorescence protein (eGFP) in both *E*. *coli* and *L*. *casei*, suggesting that P_*SlpA*_ functioned as a bifunctional promoter [20]. Here, we further tested P_*SlpA*_-driven expression in these organisms with *manB* from *B*. *pumilus* (encoding β-1,4-mannanase) and *gusA* from *E*. *coli* (encoding β-glucuronidase), the latter of which is a common reporter gene in bacterial genetics. The coding sequence of each gene was cloned downstream of P_*SlpA*_ in pELX1, yielding the expression plasmids pELX1-ManB and pELX1-GusA, which were transformed into *E*. *coli* JM109. The *E*. *coli* transformants were grown in LB broth and sampled for SDS-PAGE analysis of their total proteins. Both recombinant proteins formed discrete protein bands, suggesting that the lactobacilli promoter P_*SlpA*_ conferred a high-level of recombinant protein expression in *E*. *coli* (S1 Fig).

**Fig 1:**
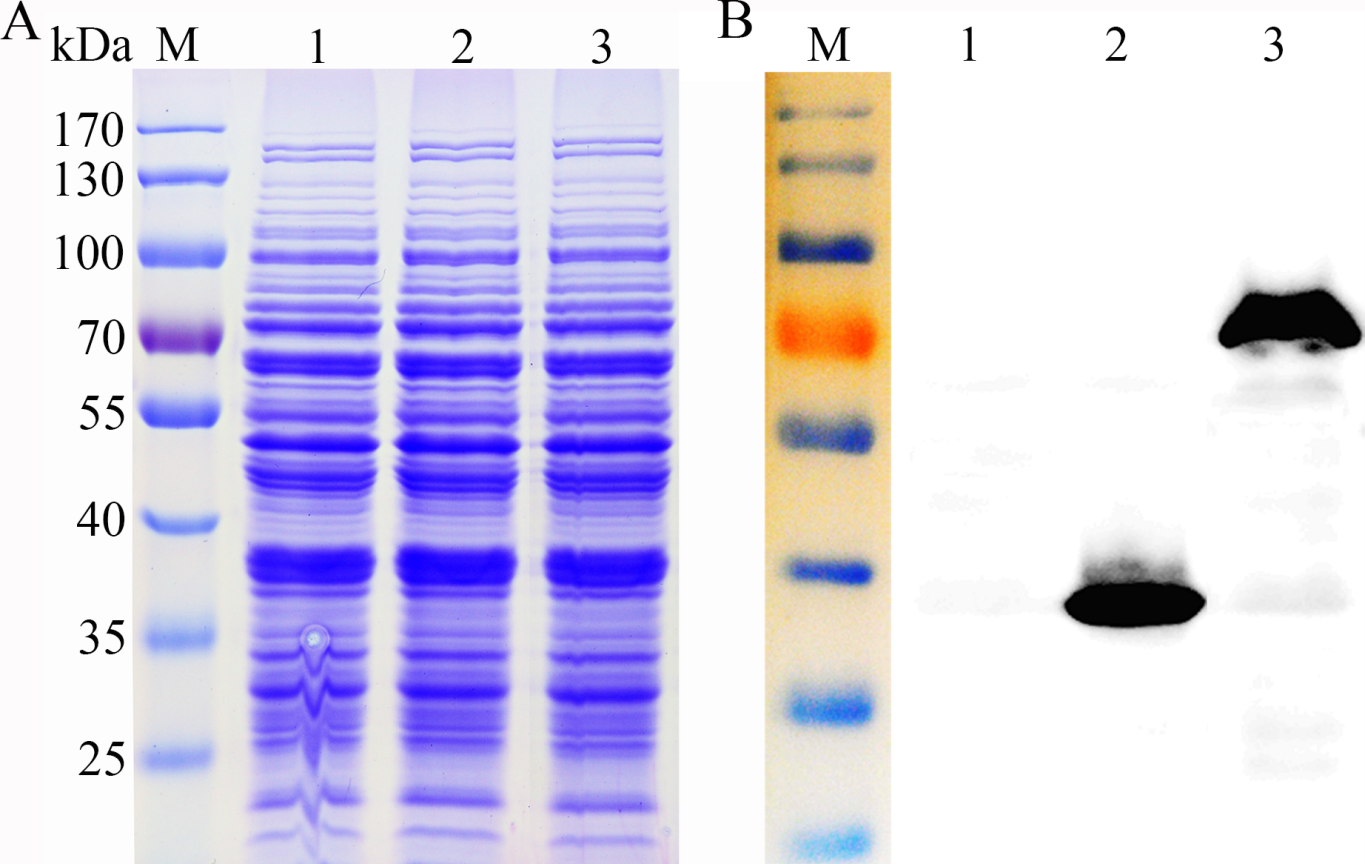
SDS-PAGE and western blot of His-tagged recombinant proteins in *L*. *casei* MCJΔ1. (**A**) SDS-PAGE analysis. Lane M, marker. Lanes 1–3, whole protein extracts of the pELX1, pELX1-ManB and pELX1-GusA transformants. (**B**) Western blot analysis, which suggested that both recombinant proteins were expressed. Lane M, marker. Lanes 1–3, whole protein extracts of the pELX1, pELX1-ManB and pELX1-GusA transformants. The protein concentrations of all samples were normalized to 3 mg·ml^-1^, and the sample volume loaded was15 μl in both the SDS-PAGE and western blot analysis.

These expression plasmids were then introduced into *L*. *casei* MCJΔ1 by electroporation, and the transformants were grown in MRS broth. Analysis of the total proteins prepared from the *L*. *casei* cultures failed to reveal any discrete bands of recombinant protein (Fig 1A), but the recombinant proteins were readily identified by western blot analysis using antisera against the His epitope (Fig 1B). These results suggest that P_*SlpA*_ did not function as a strong promoter in *L*. *casei*, although P_*SlpA*_ has been shown in several reports to function as a strong promoter in different LAB strains [28,29].

**Fig 2:**
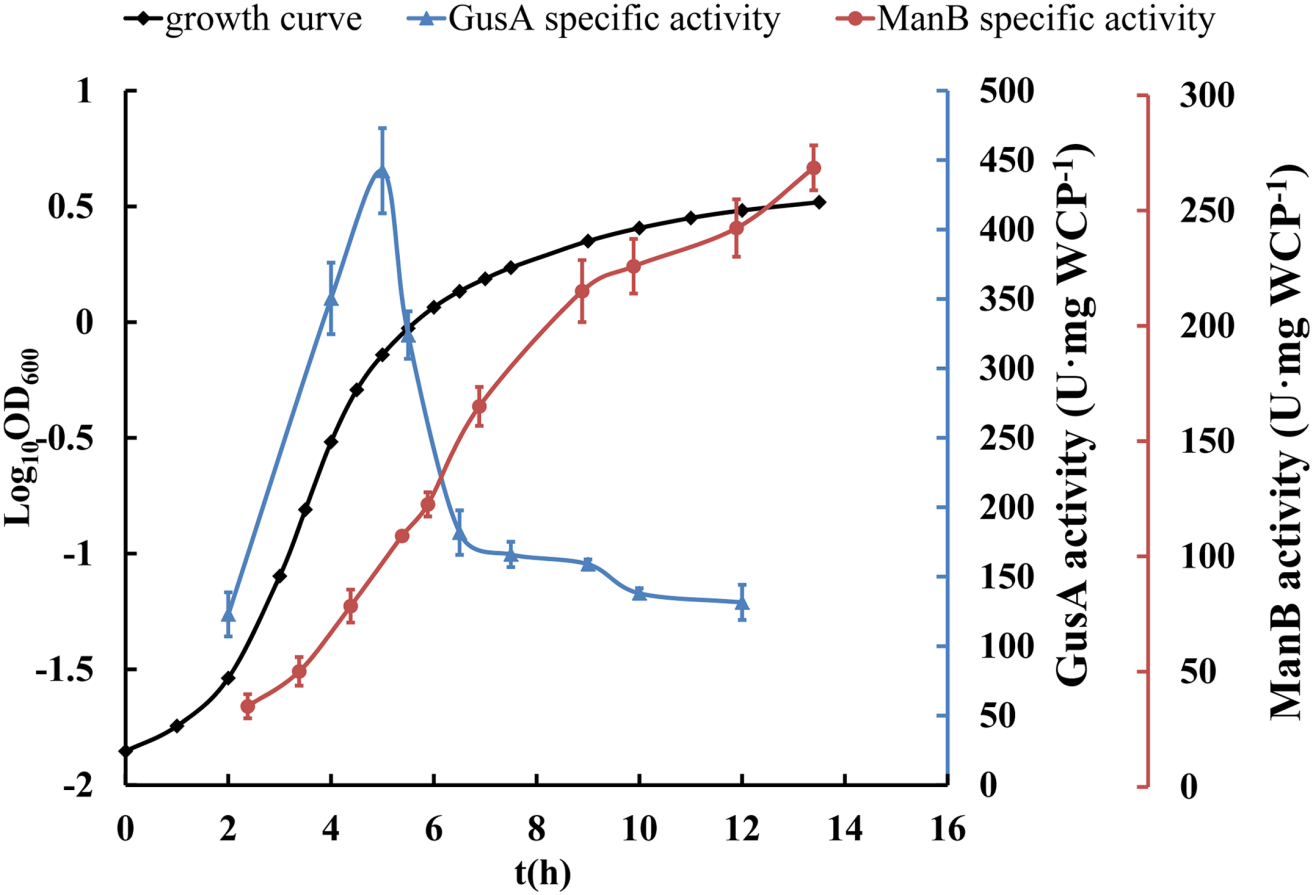
Expression of ManB and GusA in *E*. *coli* JM109. The specific activity of ManB was high and remained proportional to cell mass throughout growth, whereas GusA activity decreased severely in the late exponential growth phase (from 450 U·mg^-1^ WCP to 180 U·mg^-1^ WCP in 1.5 h) and stabilized in the stationary phase (130–180 U·mg^-1^ WCP). In the growth curve, there were no significant difference between the negative control *E*. *coli* JM109 pELX1 transformant, the *E*. *coli* JM109 ManB transformant and the *E*. *coli* JM109 GusA transformant. Error bars represent standard errors from three replicate experiments.

To investigate the detailed activities of this promoter in *E*. *coli* and in *L*. *casei*, cell extracts were prepared from exponential growth-phase cultures of both bacterial transformants and tested for the specific activity of each enzyme. The specific activity of ManB was high and remained proportional to cell mass throughout the growth of *E*. *coli*; therefore, we concluded that P_*SlpA*_ conferred a continuously high level of gene expression in *E*. *coli*. However, GusA activity in *E*. *coli* decreased severely in the late exponential growth phase and stabilized in the stationary phase (Fig 2).

**Fig 3:**
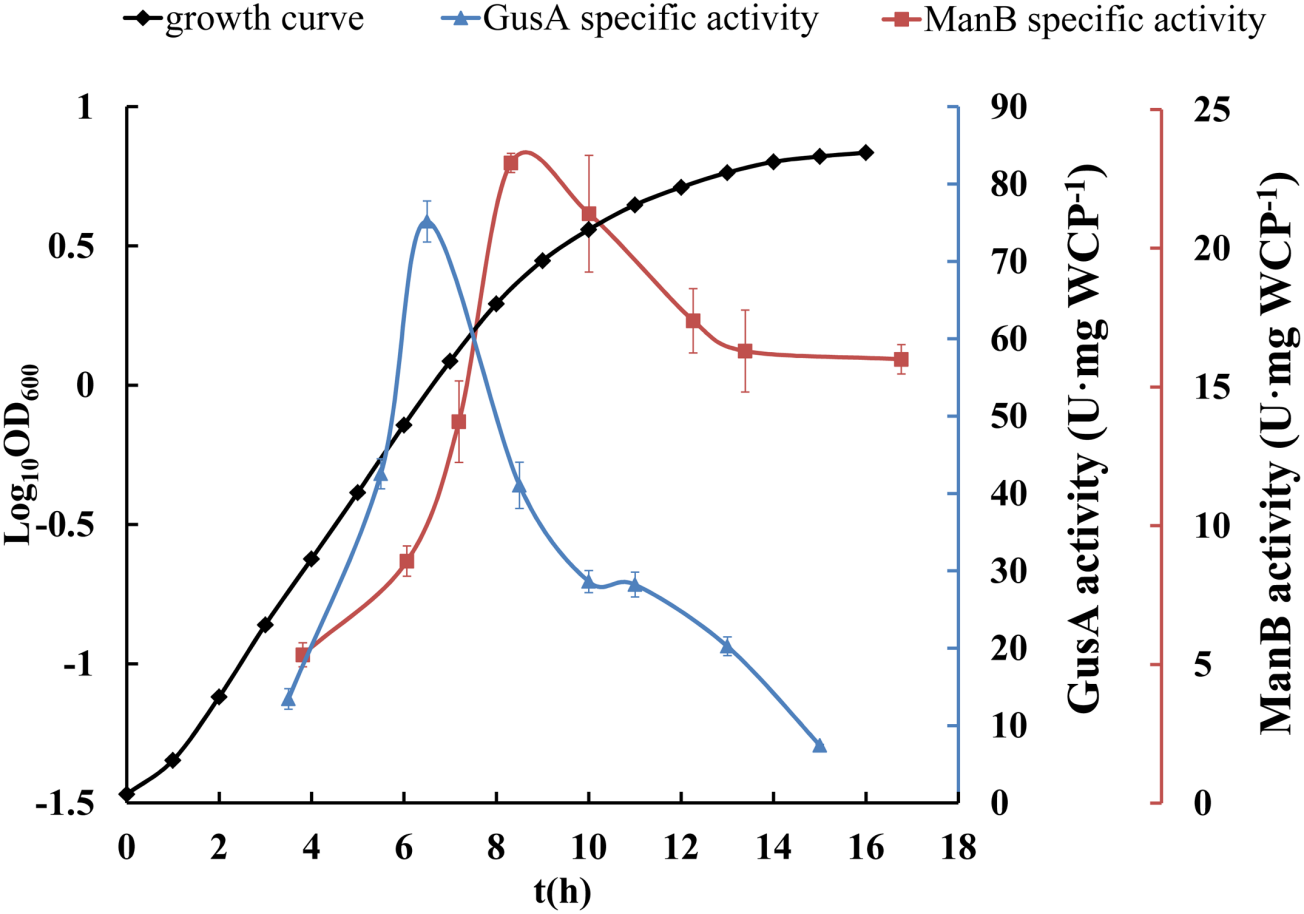
Expression of ManB and GusA in *L*. *casei* MCJΔ1. In the growth curves, *L*. *casei* MCJΔ1 showed exponential growth until OD_600_ = 1.8, and the stationary phase was from OD_600_ = 1.8 to 6.8. In the growth curve, there were no significant difference between the negative control *L*. *casei* MCJΔ1 pELX1 transformant, the *L*. *casei* MCJΔ1 ManB transformant and the *L*. *casei* MCJΔ1 GusA transformant. the OD_600_ in the activity curve of *L*. *casei* MCJΔ1/pELX1-ManB transformant were 0.2, 0.5, 1.0, 1.8, 2.9, 4.3, 5.0 and 6.7, and those of *L*. *casei* MCJΔ1/pELX1-GusA transformant were 0.2, 0.5, 1.0, 2.2, 3.6, 4.6, 5.8 and 6.6, respectively. The specific activity of ManB increased in the exponential growth phase until the OD_600_ of the culture reached 1.8 (23 U·mg^-1^ WCP). Then, ManB activity decreased gradually to 15 U·mg^-1^ WCP in the late growth phase before remaining relatively constant. The specific activity of GusA peaked during the exponential growth phase (OD_600_ = 1.0, 75 U·mg^-1^ WCP) before decreasing rapidly in the late growth phase by more than 60% (OD_600_ = 1.0 versus 4.6). Error bars represent standard errors from three replicate experiments.

In the growth curves, *E*. *coli* showed exponential growth when OD_600_<1.0, whereas *L*. *casei* maintained exponential growth until OD_600_ = 1.8 (Fig 3). We found that both reporters showed distinctive changes in *L*. *casei* during growth. The specific activity of ManB only increased in the exponential growth phase until the OD_600_ of the culture reached 1.8 (23 U·mg^-1^ WCP). Then, ManB activity decreased gradually to 15 U·mg^-1^ WCP in the late growth phase before remaining relatively constant (Fig 3). However, the specific activity of GusA peaked during the exponential growth phase (OD_600_ = 1.0, 75 U·mg^-1^ WCP) before dropping ca. 60% from the exponential growth phase to the stationary growth phase (OD_600_ = 1.0 versus 4.6) (Fig 3). The much higher expression levels of recombinant ManB and GusA in *E*. *coli* than those in *L*. *casei* are not due to P_*SlpA*_ simply functioning as a stronger promoter in *E*. *coli* than in *L*. *casei* because the plasmid copy number of pELX1 was different in these two organisms. Whereas the *E*. *coli* plasmid backbone has a copy number of 500–700 [30], pELX1 has ~5 copies per cell in *L*. *casei* [20].

As the negative control, the *L*. *casei* MCJΔ1 pELX1 transformant and the *E*. *coli* JM109 pELX1 transformant exhibited no detectable ManB activity, and the expression of ManB did not impede the growth of *L*. *casei* MCJΔ1 and *E*. *coli* JM109 hosts, thus the *manB* functioned as a good reporter gene in both bacteria. On the other hand, the *L*. *casei* MCJΔ1 pELX1 transformant exhibited no detectable GusA activity but in view of the rapid change of GusA activity during growth, which could create another layer of uncertainty in research results, thus the *gusA* maybe not a suitable reporter gene in some experiments which require enzyme assays to be performed at the exact same growth phases for all samples.

The ManB activity and cell biomass increased in early growth stages and decreased gradually in later growth stages (Fig 3). These results suggest that P_*SlpA*_ confers a high level of ManB expression in the exponential growth phase of *L*. *casei*. Furthermore, we also observed that ManB activity continuously increased throughout the growth of *E*. *coli* (Fig 2). Altogether, these results suggest that the P_*SlpA*_ promoter conferred different target gene expression levels in the two bacteria, whereas the expression from the promoter should be under stringent control in *L*. *casei* such that it might confer very low expression in the late growth phases of this bacterium, expression from this promoter in *E*. *coli* should not be inhibited.

### Protein stability and enzymatic activity of β-1,4-mannanase and β-glucuronidase in *L*. *casei*

**Fig 4:**
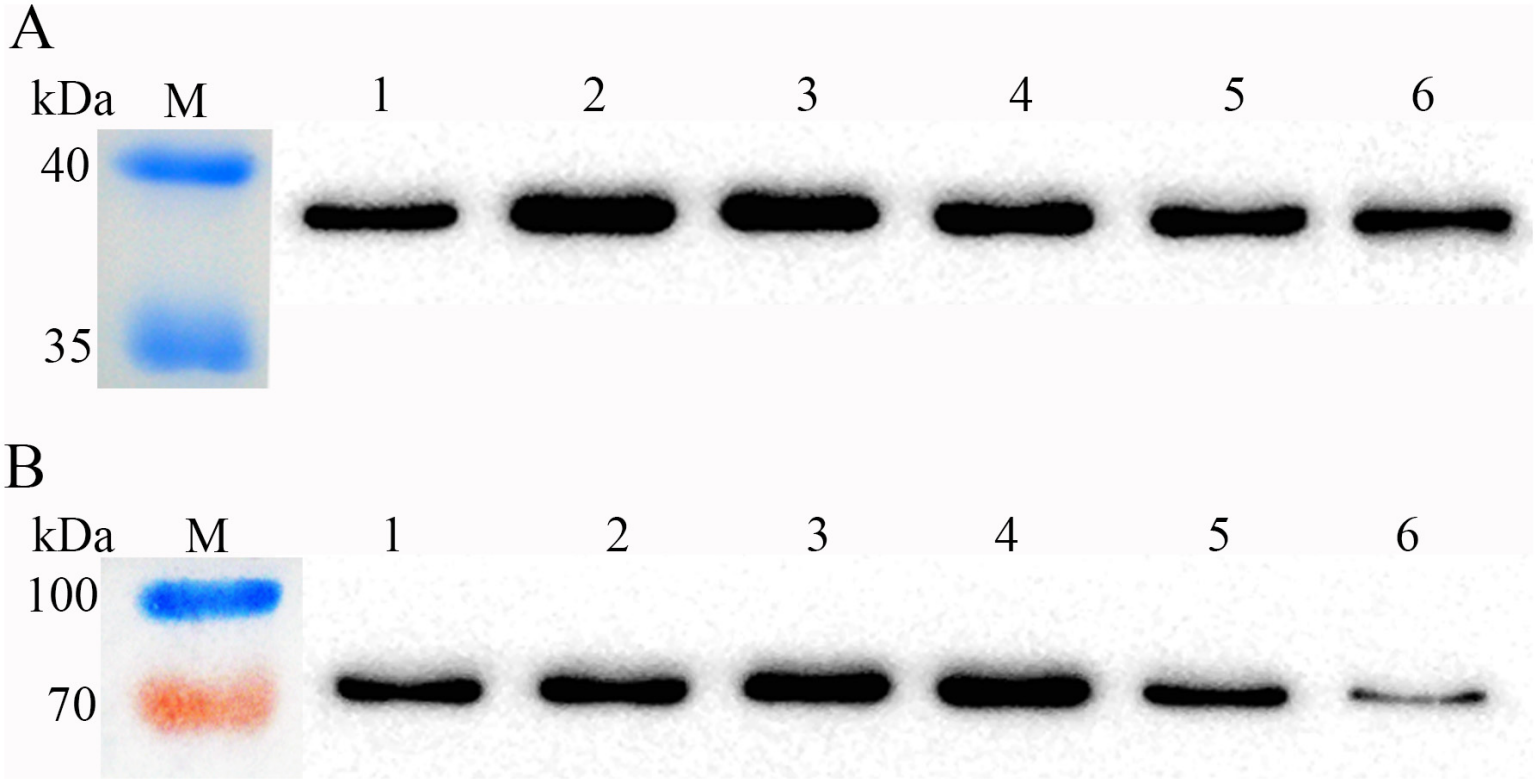
Western blot detection of ManB and GusA in *L*. *casei*. The final concentration of all the WCP samples were adjusted to 0.2 mg·ml^-1^ for ManB samples and 0.1 mg·ml^-1^ for GusA samples, and 10-μl aliquots were added in each lane of the electrophoresis gels. (**A**) Western blot of His-tagged ManB from the *L*. *casei* MCJΔ1 transformant. These results show a similar increase in the amount of ManB protein and ManB activity during early exponential growth (until OD_600_ = 1.8). Lane M, marker. Lanes 1–6, ManB from the *L*. *casei* MCJΔ1 transformant at different cell densities (OD_600_ = 1.0, 1.8, 2.9, 4.3, 5.0 and 6.7). (**B**) Western blot of His-tagged GusA from the *L*. *casei* MCJΔ1 transformant. These results show that although GusA activity decreased rapidly from the exponential growth phase to the stationary growth phase, the western blot analysis of GusA revealed large amounts of this protein in late growth-phase cell samples, when the specific activity of the enzyme dropped markedly. Lane M, marker. Lanes 1–6, GusA of the *L*. *casei* MCJΔ1 transformant at different cell densities (OD_600_ = 1.0, 2.2, 3.6, 4.6, 5.8 and 6.6).

To illustrate whether enzymatic activity reflects the level of recombinant enzyme in *L*. *casei*, cell extracts were prepared from different culture time points and used to assay ManB or GusA specific activity. Western blot analysis was also performed on these samples using antisera raised against the His epitope to estimate the amount of recombinant protein. Western blot analysis indicated similar increases in the amount of ManB protein and corresponding activity in the exponential growth phase (until OD_600_ = 1.8, Fig 4A), which suggested that the recombinant ManB was highly stable in *L*. *casei*. Furthermore, ManB activity was positively correlated with the accumulation of interest protein during cell growth (Figs 3 and 4A). These results suggest that the specific activity of ManB decreased gradually during late exponential growth because the P_*SlpA*_ promoter conferred low target gene expression; mild protein degradation in this growth phase may also have been a contributing factor.

Next, we performed similar experiments with the pELX1-GusA transformants and found that although GusA activity decreased rapidly from the exponential growth phase to the stationary growth phase, a western blot analysis of GusA revealed large amounts of this protein accumulated in late growth-phase cell samples, while the specific activity of the enzyme dropped markedly (Figs 3 and 4B). These results suggest that there may be an inactive form of GusA in *L*. *casei*. Our results revealed different properties between the common GusA reporter enzyme and ManB, indicating that *manB* is a more suitable reporter gene for studying the expression of secreted proteins in *L*. *casei* due to the high stability of its gene product, whereas *gusA* may be more useful in characterizing inducible gene expression due to the unstable feature of this enzyme in bacterial cells.

### Characterization the secretion of ManB in *L*. *casei*

**Fig 5:**
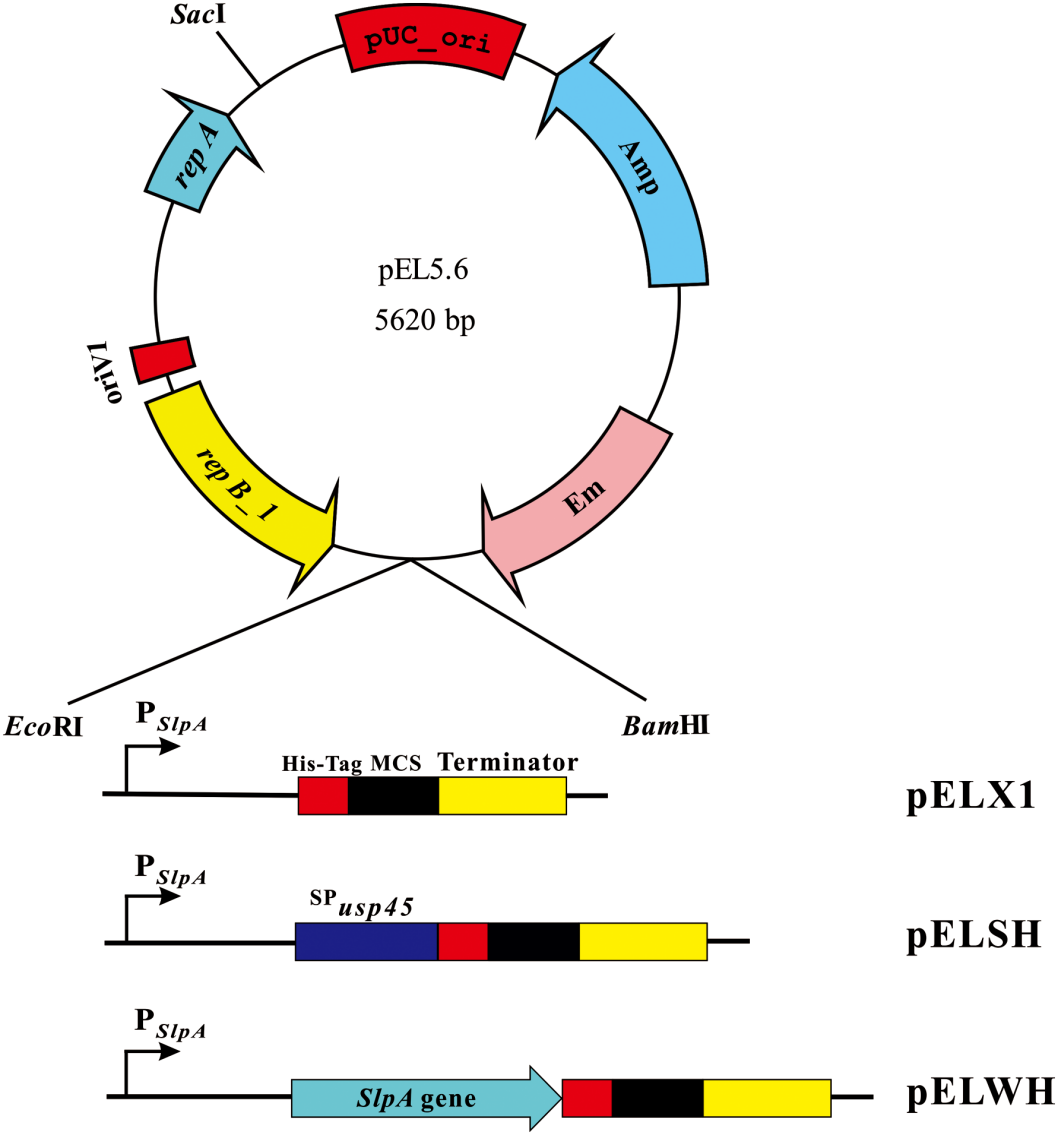
Expression cassette of related expression vectors.

ManB was employed as a reporter to study the expression of secreted protein in *L*. *casei*. *manB* was cloned into secretion expression vectors pELSH and pELWH (Fig 5) to yield pELSH-ManB and pELWH-ManB. Both secretion expression plasmids were introduced into *L*. *casei* MCJΔ1, yielding pELSH-ManB and pELWH-ManB transformants (*L*. *casei* MCJΔ1/pELSH-ManB and *L*. *casei* MCJΔ1/pELWH-ManB). These transformants were used to investigate the efficiency of these secretory elements in secreting ManB in *L*. *casei*.

**Fig 6:**
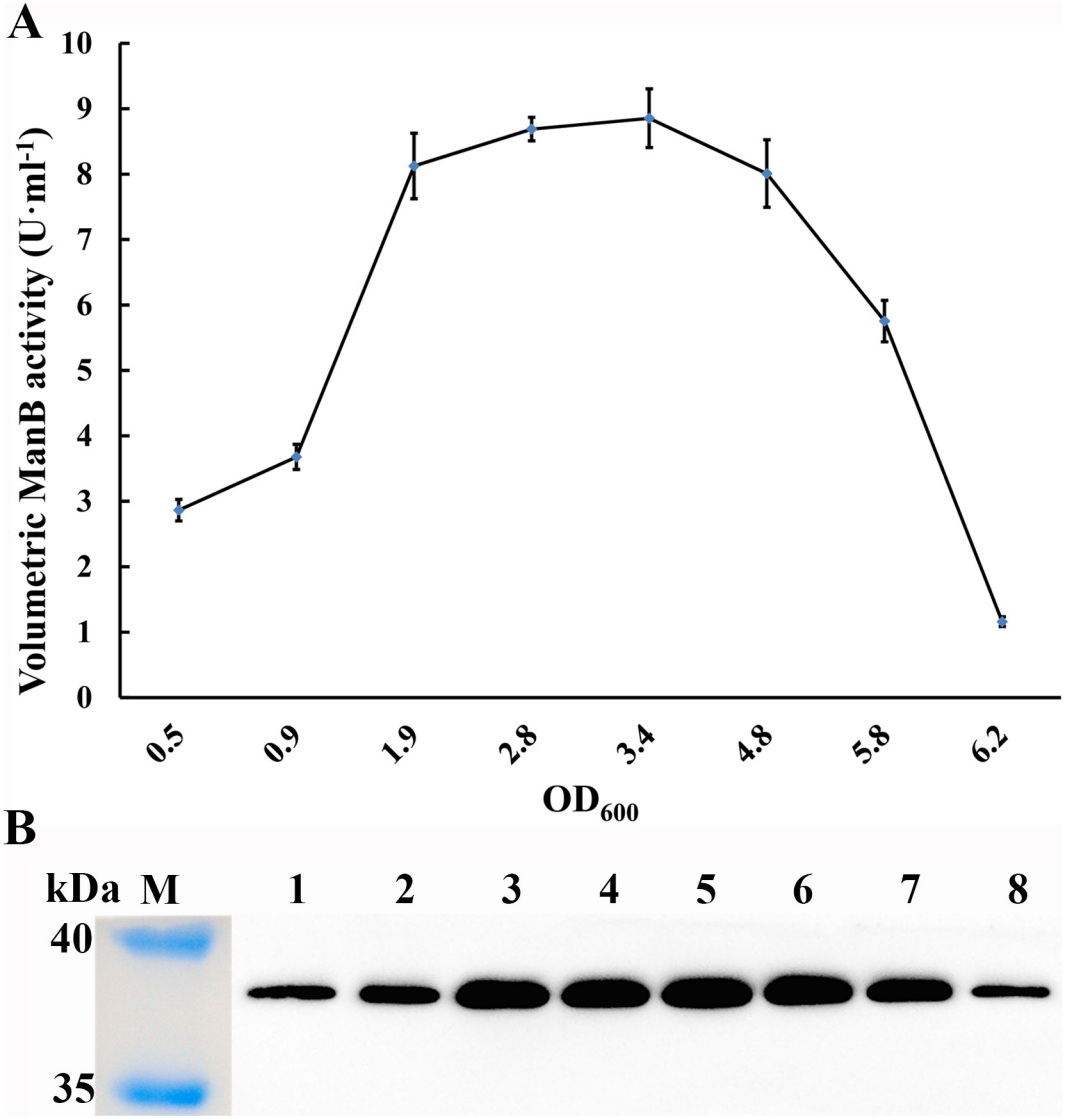
Expression and secretion of ManB in *L*. *casei* MCJΔ1/pELSH-ManB. (**A**) ManB activity in the supernatant. ManB activity reached 8.85 U·ml^-1^ during the exponential growth phase of *L*. *casei* MCJΔ1/pELSH-ManB and remained relatively constant above 8 U·ml^-1^ throughout the late growth phase (between OD_600_ = 1.9 to 4.8) before decreasing. (**B**) Western blot of His-tagged ManB in the supernatant. Aliquots of 10 μl were added in each lane of the electrophoresis gels. These results clearly showed that secreted mannanase activity was correlated with the accumulation of ManB during cell growth. Lane M, marker. Lanes 1–8, ManB of the *L*. *casei* MCJΔ1 transformant at different cell densities (OD_600_ = 0.5, 0.9, 1.9, 2.8, 3.4, 4.8, 5.8 and 6.2). Error bars represent standard errors from three replicate experiments.

The secretion of ManB reached 8.85 U·ml^-1^ in supernatant during the exponential growth phase of *L*. *casei* MCJΔ1/pELSH-ManB and remained relatively constant above 8 U·ml^-1^ in supernatant throughout the late growth phase between OD_600_ = 1.9 to 4.8 (Fig 6A). Western blot analysis clearly showed that mannanase activity was correlated with the secretion of ManB during cell growth (Fig 6B). The *L*. *casei* MCJΔ1/pELX1-ManB transformant was precessed by the same method, but no ManB activity and recombinant ManB protein were detected. These results togetherly indicated ManB is secreted using the SP_Usp45_ as secretion signal in *L*. *casei* MCJΔ1.

**Fig 7:**
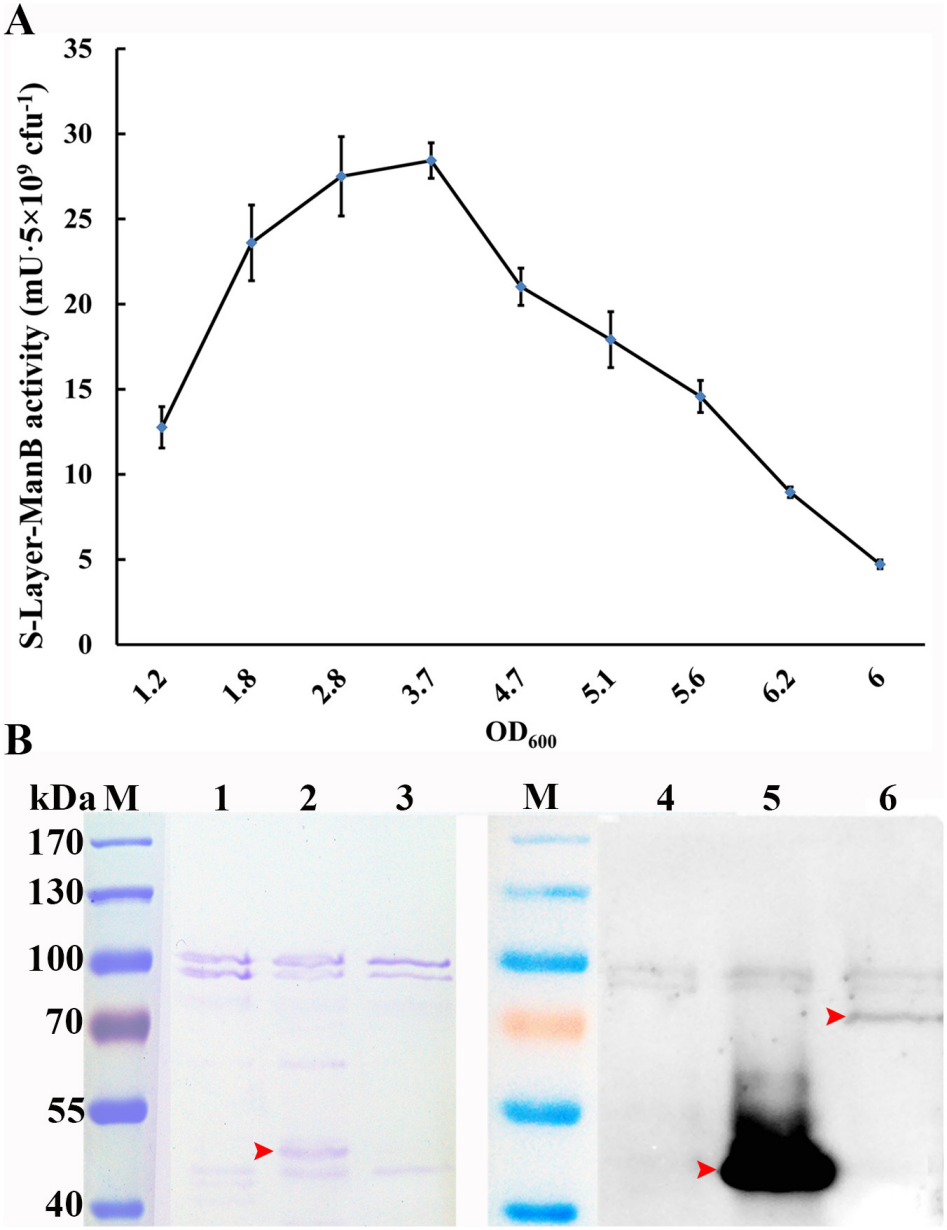
Expression and secretion of the ManB-SlpA fusion protein in *L*. *casei* MCJΔ1/ pELWH-ManB. **(A)** S-layer-bound mannanase activity from the functional fusion protein on the cell surface of *L*. *casei* MCJΔ1/pELWH-ManB. The mannanase activity increased during the early growth phase, peaking at OD_600_ = 3.7 with an activity of 28.4 mU·5×10^9^ cfu^-1^, and then decreased. **(B)** SDS-PAGE and western blot analysis of the composition of the S-layer proteins. *L*. *casei* MCJΔ1 and *L*. *casei* MCJΔ1/pELWH were used as controls (the OD_600_ of all cell samples was 3.0). The fusion protein was barely detectable in *L*. *casei* MCJΔ1/pELWH-ManB (Lane 6), as reflected by a very low mannanase activity on the cell surface. Lane M, Marker. Lanes 1–3, SDS-PAGE-analyzed S-layer proteins from *L*. *casei* MCJΔ1, *L*. *casei* MCJΔ1/pELWH and *L*. *casei* MCJΔ1/pELWH-ManB. Lanes 4–6, western blot of His-tagged target proteins from the S-layer proteins of *L*. *casei* MCJΔ1, *L*. *casei* MCJΔ1/pELWH and *L*. *casei* MCJΔ1/pELWH-ManB. The red arrow represents the target proteins. Error bars represent standard errors from three replicate experiments.

For the *L*. *casei* MCJΔ1/pELWH-ManB transformant, as expected, ManB activity was detected on the cell surface of contact bacteria. This activity peaked at OD_600_ = 3.7 with an activity of 28.4 mU·5×10^9^ cfu^-1^ (Fig 7A). Furthermore, the S-layer proteins of *L*. *casei* MCJΔ1/pELWH-ManB were analyzed by SDS-PAGE and western blot, using *L*. *casei* MCJΔ1 and *L*. *casei* MCJΔ1/pELWH as controls. A considerable amount of SlpA accumulated in the S-layer of *L*. *casei* MCJΔ1/pELWH (Fig 7B, Lanes 2 and 5). In contrast, the fusion protein was barely detectable in *L*. *casei* MCJΔ1/pELWH-ManB (Fig 7B, Lane 6), as reflected by a very low mannanase activity on the cell surface.

## Discussion

LAB exhibit several advantages over other microorganisms as microbial hosts for heterologous gene expression. LAB are usually regarded as GRAS organisms, and they have a long history of sustainable, stable and generally safe use in the production of fermented food. Some of them are considered to be beneficial probiotic bacteria [31], thus making them ideal hosts for food-grade gene expression. However, the low protein expression efficiency of known LAB expression systems is a major bottleneck for this application. While a common approach for increasing the yield of recombinant proteins is to identify promoters of high efficiency, very few studies have pursued this approach in LAB. One of the main reasons accounting for this lack of research is the paucity of known LAB reporter gene systems. Here we tested two bacterial genes as reporters in *L*. *casei* and investigated their expression driven by the lactobacilli *slpA* gene promoter in an expression vector recently developed by our laboratory. We found both genes, *B*. *pumilus manB* and *E*. *coli gusA*, could be used as reporter genes in both *L*. *casei* and *E*. *coli*. We also found that the two reporter genes exhibit distinct properties: GusA accumulates in an inactive form by a yet unknown mechanism, whereas ManB is highly active and only becomes inactivated by protein degradation.

Several other genes have been used as reporters in LAB genetics. For example, *eGFP* was used as a reporter gene in *L*. *casei* [18,32]. However, although the detection of protein in this reporter system is simple, requiring only irradiation with blue light and analysis by fluorescence microscopy, the system is not suitable for studying promoter activities because protein quantification is technically difficult [19]. *E*. *coli gusA* provides an alternative approach. However, the rapid change of reporter gene activity during growth requires enzyme assays to be performed at the exact same growth phases for all samples, which could create another layer of uncertainty in research results. For example, a previous study indicated that the level of GusA activity was ten-fold higher in exponential-phase cultures than in stationary-phase cultures when expressed from six constitutive promoters in both *L*. *casei* and *Lc*. *lactis* [33].

Here we report that ManB functions as a good gene reporter in *L*. *casei* and *E*. *coli* systems. The gene codes for a β-1,4-mannanase that catalyzes mannan hydrolysis, releasing manno-oligosaccharides. Many microorganisms can utilize mannan as a carbon source, including *Bacillus* spp. Neither *E*. *coli* JM109 nor *L*. *casei* MCJΔ1 showed detectable mannanase activity; therefore, *manB* can function as a reporter gene in these organisms to reveal promoters with low activities. Furthermore, the ManB protein is very stable and maintains its active form *in vivo*, which is consistent with reports showing that the protein is highly active and remarkably thermostable at 60°C [34–36]. Here, we further show that *manB* is stable after secretion into the culture medium and is only subjected to degradation in the stationary growth phase. Therefore, *manB* constitutes a good reporter gene for studying the expression of secreted proteins in *L*. *casei*.

Furthermore, our work also indicates that the lactobacilli P_*SlpA*_ promoter confers different expression levels in *L*. *casei* and *E*. *coli*. The most likely interpretation of these results is that this promoter directs stringent growth phase-dependent expression in homologous hosts but becomes a constitutive promoter in *E*. *coli*. This conclusion may indicate the requirement of a transcriptional repressor that is common in homologous hosts but absent from *E*. *coli*. This finding has two implications for LAB research. First, previously identified strong bacterial promoters should be assayed in LAB to examine their activities. Second, as *E*. *coli* genetics is more straightforward, preliminary screening in *E*. *coli*, followed by re-testing the best candidates in other bacteria, may facilitate more efficient research. Finally, we have demonstrated that *manB* is a more robust reporter gene than *gusA* for *L*. *casei* genetic applications, and this reporter also shows great potential for use in the molecular genetics of other bacteria.

## Supporting Information

S1 Fig SDS-PAGE analysis of ManB and GusA protein expression by *E*. *coli* JM109. Lane M, marker. Lanes 1–3, whole protein lysates of pELX1, pELX1-ManB and pELX1-GusA transformants. Lane 4, nickel-affinity purification of His-tagged ManB with 200 mM imidazole. Lane 5, nickel-affinity purification of His-tagged GusA with 200 mM imidazole. The red arrow represents the target proteins.
(TIF)
