# Supplementary figures and images for: Heterologous Expression of Mannanase and Developing a New Reporter Gene System in Lactobacillus casei and Escherichia coli
Source: PLoS One. 2015 Nov 12;10(11):e0142886. doi: 10.1371/journal.pone.0142886 (PMC4643024; doi:10.1371/journal.pone.0142886)

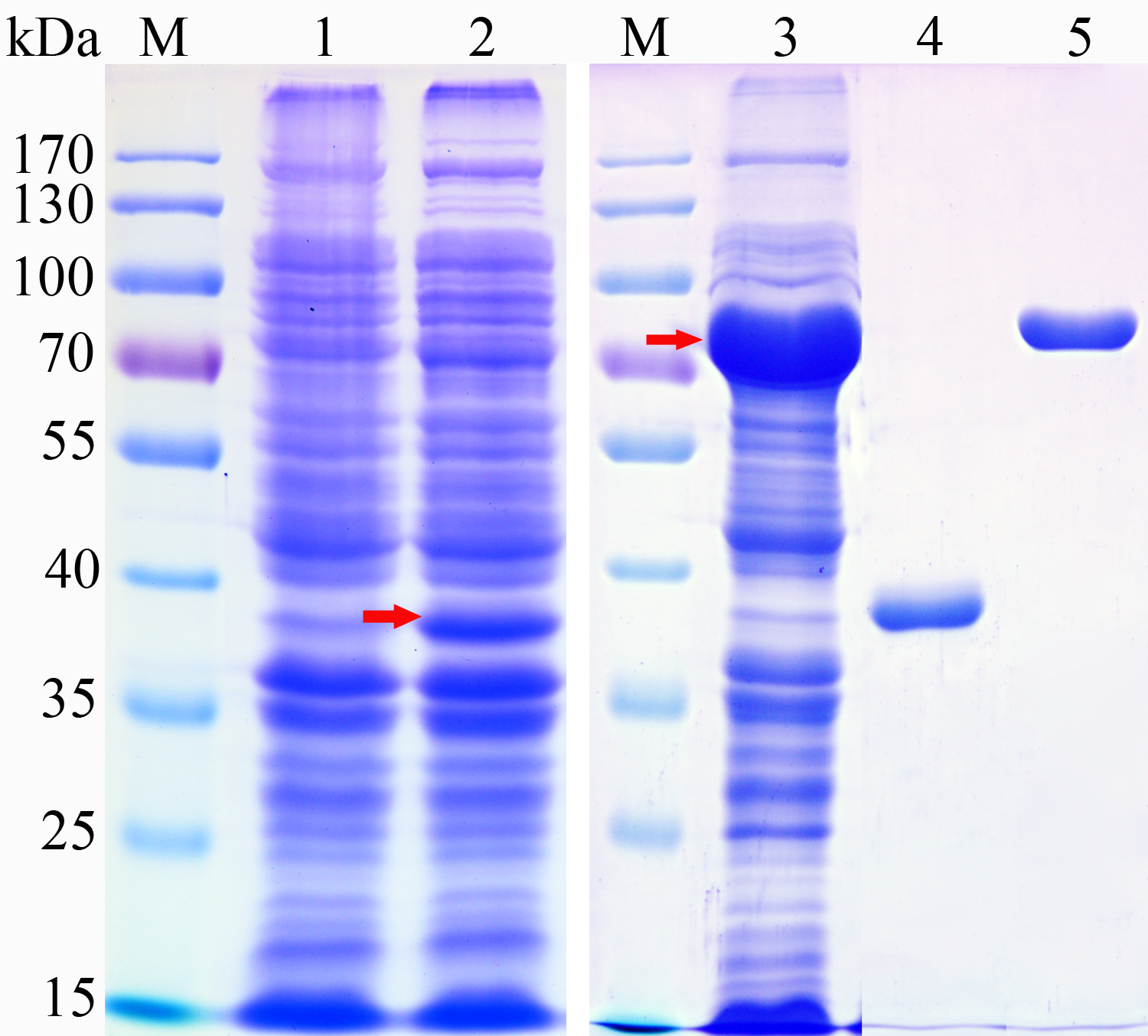

Supplement: S1 Fig — Lane M, marker. Lanes 1–3, whole protein lysates of pELX1, pELX1-ManB and pELX1-GusA transformants. Lane 4, nickel-affinity purification of His-tagged ManB with 200 mM imidazole. Lane 5, nickel-affinity purification of His-tagged GusA with 200 mM imidazole. The red arrow represents the target proteins. (TIF) [file pone.0142886.s001.tif]
